# Four Japanese Patients with Congenital Nephrogenic Diabetes Insipidus due to the *AVPR2* Mutations

**DOI:** 10.1155/2018/6561952

**Published:** 2018-07-03

**Authors:** Noriko Namatame-Ohta, Shuntaro Morikawa, Akie Nakamura, Kumihiro Matsuo, Masahide Nakajima, Kazuhiro Tomizawa, Yusuke Tanahashi, Toshihiro Tajima

**Affiliations:** ^1^Department of Pediatrics, Hokkaido University Graduate School of Medicine, North 15 West 7, Kita-ku, Sapporo 060-8638, Japan; ^2^Department of Pediatrics, Asahikawa City Hospital, 1-1-65 Kinsei-cho, Asahikawa 070-8610, Japan; ^3^Department of Molecular Endocrinology, National Research Institute for Child Health and Development, 2-10-1 Okura, Setagaya-ku, Tokyo 157-8535, Japan; ^4^Department of Pediatrics, Asahikawa Medical University, 2-1-1-1 Midorigaoka-higashi, Asahikawa 078-8510, Japan; ^5^Department of Pediatrics, Nakashibetsu Town Hospital, West 10 South 9, Nakashibetsu 086-1110, Japan; ^6^Department of Pediatrics, Jichi Children's Medical Center Tochigi, 3311-1 Yakushiji, Shimotsuke 329-0498, Japan

## Abstract

Almost 90% of nephrogenic diabetes insipidus (NDI) is caused by mutations in the arginine vasopressin receptor 2 gene (*AVPR2*) on the X chromosome. Herein, we reported clinical and biochemical parameters in four cases of three unrelated Japanese families and analyzed the status of the *AVPR2.* Two of the four patients had poor weight gain. However, in the male and female sibling cases, neither had poor weight gain while toddlers, but in the male sibling, episodes of recurrent fever, polyuria, and polydipsia led to the diagnosis of NDI at 4 years of age. Analysis of *AVPR2* identified two nonsense mutations (c.299_300insA; p.K100KfsX91 and c.296G > A; p.W99X) and one missense mutation (c.316C > T; p.R106C). These mutations were previously reported. The patient with c.316C > T; p.R106C had milder symptoms consistent with previous reports. Of the familial cases, the sister was diagnosed as having NDI, but a skewed X-inactivation pattern in her peripheral blood lymphocytes was not identified. In conclusion, our study expands the spectrum of phenotypes and characterized mutations in *AVPR2* in NDI.

## 1. Introduction

Nephrogenic diabetes insipidus (NDI) is a rare disease that is characterized by resistance of the distal renal tubule and collecting ducts to arginine vasopressin [1, 2]. Vast majority of NDI is caused by mutations in the arginine vasopressin receptor 2 gene (*AVPR2*) on the X chromosome [3]. At present, more than 250 mutations have been reported [2]. Mutations in *AVPR2* were classified into three types. Type-I mutants reach the cell surface but cannot bind its ligand, type-II mutant receptors have impaired intracellular transport and cannot reach the cell surface, and type-III mutants are inappropriately transcribed [4, 5].

Common symptoms in male patients are polyuria, polydipsia, fever of unknown etiology, convulsions, and vomiting, which usually develop soon after birth [6]. On the other hand, female cases have only mild symptoms [7]. Furthermore, some mutations in the *AVPR2* are related to partial NDI [8, 9].

In this study, we assessed the clinical and biochemical parameters and *AVPR2* status in four NDI cases of three unrelated Japanese families.

## 2. Subjects and Methods

### 2.1. Subjects

Clinical symptoms, age at diagnosis, biochemical data, and current treatment are summarized in Table 1. All four patients had polyuria and polydipsia, and results of biochemical evaluations showed high plasma antidiuretic hormone (ADH) levels. Based on these findings, NDI was suspected. The Institutional Review Board Committee of Hokkaido University approved this study (approval number 13-061). The patients' parents provided written informed consent for their children's participation in this study.

#### 2.1.1. Case 1

A 3-month-old Japanese boy was admitted because of poor body weight gain, vomiting, and fever that had persisted for one week. He was born as a full-term infant with no complications during pregnancy.

**Figure 1:**
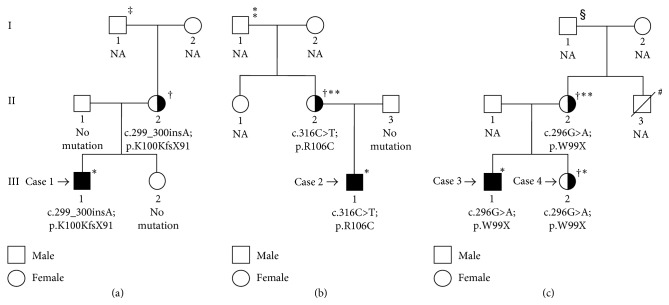
Family trees of 4 Japanese patients with congenital nephrogenic diabetes insipidus due to the *AVPR2* mutations. Family trees of (a) Case 1, (b) Case 2, and (c) Cases 3 and 4. Index cases, also indicated by arrows, are represented by filled boxes and carriers as half-filled circles. ^†^X-inactivation patterns were analyzed; ^*∗*^polydipsia and polyuria; ^*∗∗*^mild polydipsia; ^‡^maternal grandfather of Case 1 had tendency of polydipsia, but the detail is not clear; ^⁑^as maternal grandfather of Case 2 is dependent on alcohol, and polydipsia and polyuria are not clear; ^§^maternal grandfather of Cases 3 and 4 had a tendency of polydipsia, but the detail examination is not performed; ^#^maternal uncle of Cases 3 and 4, who had been undergone artificial dialysis for renal failure, died at the age of forty-seven. The detail for renal failure was not clear; NA, not accessed.

At the time of admission, he had polyuria with a urine volume of 700–800 mL/d. Results of laboratory examinations are shown in Table 1. Findings of brain magnetic resonance imaging (MRI) were normal. Based on the polyuria and the high serum ADH level, the infant was diagnosed as having NDI, and hydrochlorothiazide was initiated. Spironolactone and potassium supplementation was added when he was 2 years old and 4 years old, respectively, and indomethacin and a protein-restricted diet were initiated when he was 6 years old. He is currently 13 years old. His height is 150 cm (−0.8 SD), and his weight is 37 kg (−0.6 SD). His urine volume is approximately 7 L/day. He has mild hydronephrosis in the right kidney. His mother is asymptomatic. The family tree of Case 1 is shown in Figure 1.

#### 2.1.2. Case 2

In Case 2, poor weight gain was pointed out at the age of 4 months in this male Japanese infant. Polydipsia and polyuria were noted when he was 17 months of age. At that time, his water intake volume was approximately 3 L/d. Previously, he had experienced recurrent mild to moderate fevers of unknown etiology.

The laboratory examinations results are shown in Table 1. The water deprivation test showed elevated serum Na^+^, plasma osmolality, and urine osmolality (Table 2). However, the subcutaneous injection of vasopressin did not greatly increase urine osmolality. Six and a half hours after the test started, his body weight was reduced by 4.1%. Finally, his plasma ADH elevated to 110.1 pmol/L. Brain MRI findings were normal. Based on these findings, a diagnosis of partial NDI was confirmed when he was 19 months of age. Trichlormethiazide was initiated in combination with spironolactone and sodium restriction. This treatment has successfully decreased the patient's urine volume and water intake, and his body weight has caught up to near normal for his age. Now, he is 3 years old, and his height is 90.8 cm (−0.6 SD) and weight is 12.9 kg (−0.4 SD). His mother had also complained of mild polydipsia (2,000 mL/day) and polyuria from childhood, and her plasma ADH level was mildly elevated (5.90 pmol/L), but further examination has not been done. The pedigree of this family is shown in Figure 1.

#### 2.1.3. Cases 3 and 4

Case 3 is now a 14-year-old Japanese boy. Polydipsia and polyuria were noticed at 4 years of age. He had enuresis every day from infancy. Since he had an elevated plasma ADH level (53.1 pmol/L), he was diagnosed as having NDI. The laboratory data at the time of diagnosis are shown in Table 1. He is being treated with hydrochlorothiazide, potassium supplementation, and indomethacin. Currently, his water intake is approximately 3 L/d. Case 4 is the younger sister of Case 3 and she is now 12 years old. Polydipsia and polyuria were noted when she was 4 years old after the diagnosis of NDI in Case 3. Her plasma ADH level (6.2 pmol/L) was also elevated. She was diagnosed as having NDI, and treatment with hydrochlorothiazide, potassium supplementation, and indomethacin was initiated. Their mother also complained polydipsia and polyuria since her childhood. However, her latest daily urine volume (2,000 mL/day) did not meet the diagnostic criteria for NDI. The family tree is shown in Figure 1.

### 2.2. Sequence Analysis of *AVPR2* and Study of X Chromosome Inactivation

Genomic DNA was extracted from peripheral blood leukocytes of the cases and female carriers. The *AVPR2* exon was amplified by polymerase chain reaction (PCR) using the primers as reported previously [10], and PCR products were purified and sequenced directly using an Applied Biosystems 3130 Genetic Analyzer (Applied Biosystems, Foster City, CA, USA). The X-inactivation patterns of female carriers were investigated by studying the polymorphic trinucleotide (CAG) repeats in the first exon of the human androgen receptor gene as reported previously [11].

## 3. Results

**Figure 2:**
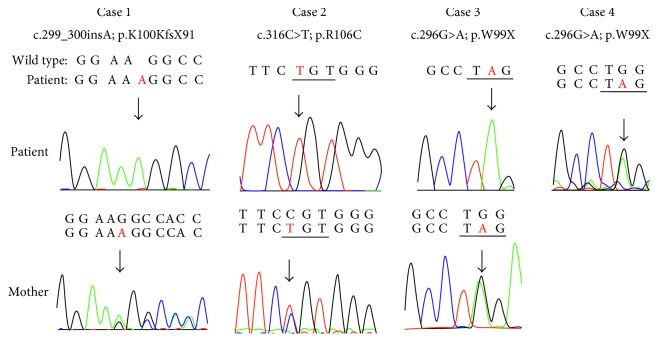
Results of sequencing of *AVPR2* mutations. Sequence chromatograms of *AVPR2* in patients and their mothers. Arrows indicate mutation sites.

Hemizygous mutations of *AVPR2* were identified in all three male patients (Figure 2). In Case 1, one base insertion caused a frame shift, generating a premature stop codon at codon 191 in exon 2 (c.299_300insA; p.K100KfsX91, designated as p.K100KfsX91). His mother was heterozygous for this mutation. Case 2 had a c.316C > T; p.R106C (designate as p.R106C) in exon 2, which was previously reported [10, 12–17]. His mother was heterozygous for this mutation. Cases 3 and 4 had a nucleotide change of G to A at position 296, resulting in the nonsense substitution (c.296G > A; p.W99X, designated as p.W99X). Their mother and Case 4 were heterozygous for the mutation. These mutations were previously reported.

**Figure 3:**
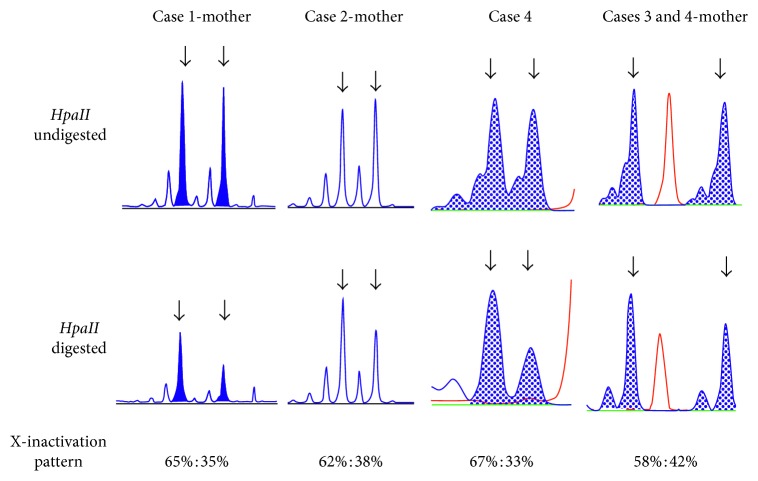
Analysis of X-chromosome inactivation. Arrows indicate the peak fluorescence intensity of the androgen CAG repeat on each X chromosome. Samples were treated with or without *HpaII*, and fluorescence intensities between treated and untreated samples were compared. The calculated X-inactivation percentage is shown at the bottom of each column.

The values of relative X-inactivation for the normal allele in Case 4 was 67.0%, and the values of mothers of all four patients were 65.0% (Case 1 mother), 62.0% (Case 2 mother), and 58.0% (Case 3 and 4 mother), respectively (Figure 3). Skewed X-inactivation is defined as inactivation of 75–80% of cells in the same allele [18]. Therefore, they had random X-inactivation.

## 4. Discussion

Currently, over 250 mutations in the *AVPR2* have been described as the cause of NDI [2]. In our study, three previously reported mutations (p.K100KfsX91, p.W99X, and p.R106C) were identified [12, 17]. The mutations p.K100KfsX91 and p.W99X produced a premature stop codon, resulting in a truncated protein. Regarding p.R106C, that mutation was identified in 7.7% (5/65) of Japanese NDI patients [17] and also was identified in other Asian and ethnic populations [2, 12, 15, 16]. The p.R106C mutation occurs at CpG nucleotides, which are mutation hot spots for genetic diseases.

Although most cases of NDI are diagnosed within the first year of life, some are diagnosed later because of milder symptoms. Especially, several missense mutations have been related to mild phenotypes of NDI [14, 17, 19]. Among our cases, Case 2 with p.R106C had poor weight gain from 4 months of age. Although he did not develop severe dehydration, he had frequent episodes of mild to moderate fever until the diagnosis was made. Pediatricians should keep in mind the possibility of mild NDI during the differential diagnosis of fever with unknown etiology.

As a previous *in vitro* study showed that p.R106C retained a slight capacity for production of cAMP in response to AVP, p.R106C is thought to cause less severe NDI [15]. Two patients with p.R106C reported by Pasel et al. [14] had high basal urine osmolality and partial response to AVP administration. Chen et al. [15] also reported that an NDI patient with p.R106C had normal urine and plasma osmolality and plasma electrolytes, similar to our patient (Case 2).

It is thought that the development of NDI in female carriers is due to skewed X-chromosome inactivation of the normal allele. In one Japanese study, 25% of female carriers developed NDI [20]. A recent Spanish study showed a frequency of NDI in female carriers of 50% [10]. In our studies, skewed X-inactivation was not found in Case 4. This can be explained by different degrees of X-inactivation ratios in each organ as suggested previously [21, 22].

In conclusion, we report Japanese NDI patients with *AVPR2* mutations (p.K100KfsX91, p.W99X, and p.R106C). There are broad phenotypic differences among the patients with the same type of mutations. In female patients, skewed X-inactivation is not always detected in peripheral blood lymphocytes. As some NDI cases do not show severe dehydration, they should be checked not only electrolyte but also their urine and serum osmolality once they suspected NDI. Low-grade fever and poor body weight gain in infant can be the clue of diagnosing mild NDI.

## Figures and Tables

**Table 1:** Clinical characteristics of 4 patients with congenital nephrogenic diabetes insipidus and *AVPR2* mutations.

	Case 1	Case 2	Case 3	Case 4
Sex	Male	Male	Male	Female
*AVPR2* mutation	c.299_300insA; p.K100KfsX91	c.316C > T; p.R106C	c.296G > A; p.W99X	c.296G > A; p.W99X
Age^*∗*^	3 mo	19 mo	4 y	4 y
Symptoms^*∗*^	Polydipsia, polyuria, poor body weight gain, vomiting, fever	Polydipsia, polyuria, poor body weight gain, low-grade fever	Polydipsia, polyuria	Polydipsia, polyuria
*Laboratory data* ^*∗*^				
Serum Na (mEq/L)	162	139	138	141
ADH^*∗∗*^ (pmol/L)	29.4	12.7	53.1	6.2
Plasma osmolality (mOsm/L)	325	280	278	283
Urine osmolality (mOsm/L)	183	74.0	51.0	175
Urologic complications	Mild hydronephrosis (right kidney)	Calcification (right kidney), mild hydronephrosis (left kidney)	None	None
Current treatment	HCTZ SP potassium supplement IDM	TCM SP sodium restriction	HCTZ potassium supplement IDM	HCTZ potassium supplement IDM

^*∗*^Time of diagnosis; ^*∗∗*^normal range: 0.9–4.6 pmol/L. ADH, plasma antidiuretic hormone; HCTZ, hydrochlorothiazide; SP, spironolactone; TCM, trichlormethiazide; IDM, indomethacin.

**Table 2:** Results of the water deprivation test in Case 2.

Test time (hour)	Body weight (g)	Body weight loss (%)	Urine osmolality (mOsm/L)	Serum osmolality (mOsm/L)	Serum Na^+^ (mEq/L)	ADH (pmol/L)
0	9.055		68	286	140	64.7
1	8.980	0.8	150			
2	8.905	1.6	291			
3	8.855	2.2	261			
4	8.795	2.8	252			
5^*∗*^	8.745	3.4	314	292	146	91.8
6			384			
6.5	8.675	4.1	378	295	146	110.1

^*∗*^Vasopressin was subcutaneously injected. ADH, plasma antidiuretic hormone.
